# Supplementary material for: Structure of the Rpn13-Rpn2 complex provides insights for Rpn13 and Uch37 as anticancer targets
Source: Nat Commun. 2017 Jun 9;8:15540. doi: 10.1038/ncomms15540 (PMC5494190; doi:10.1038/ncomms15540)
Supplement: Supplementary Information — Supplementary figures and supplementary tables. [file ncomms15540-s1.pdf]

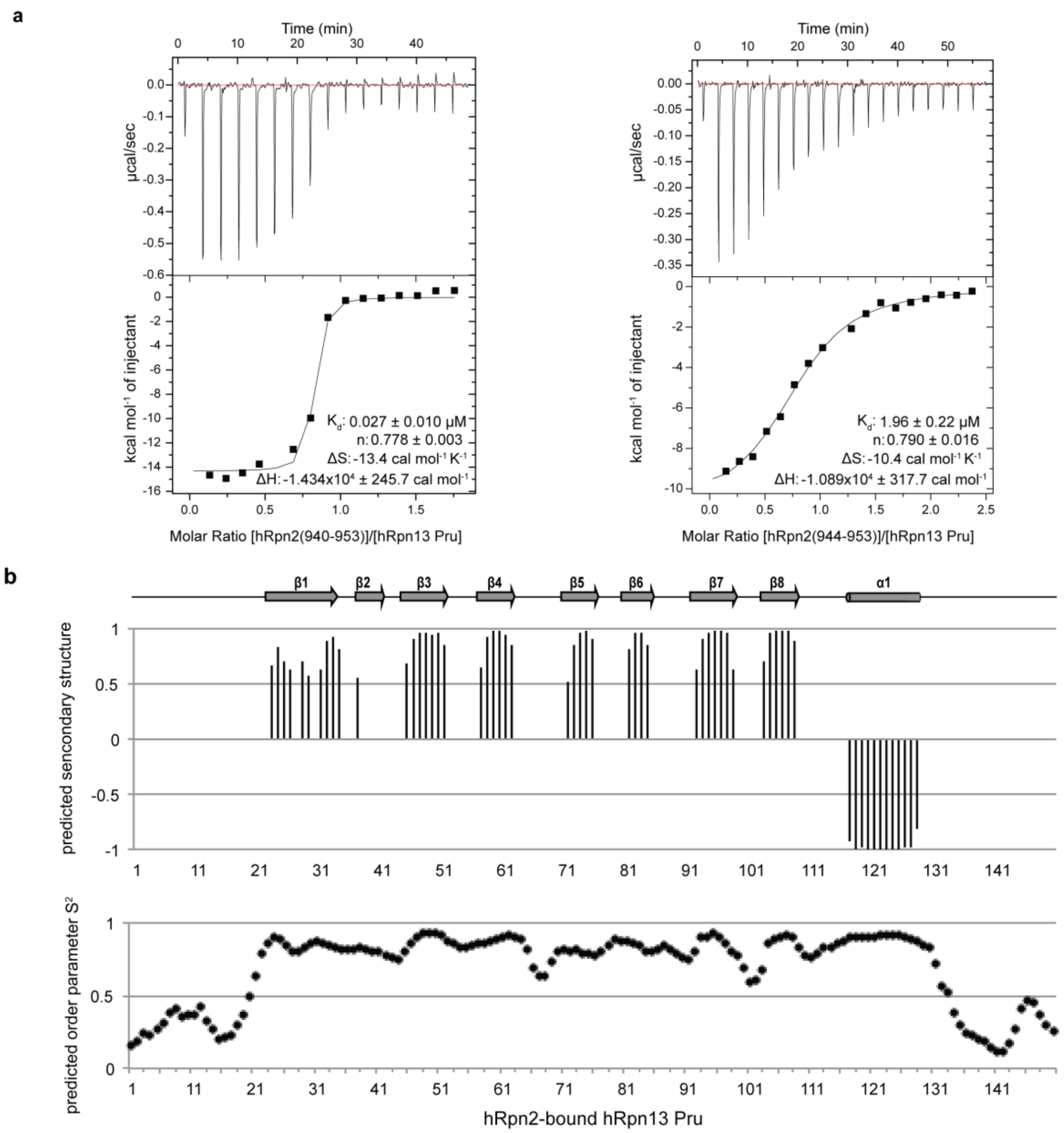

Supplementary Figure 1: The C-terminal 14 amino acids of hRpn2 bind to hRpn13 Pru with 27 nM binding affinity. (**a**) 200 μM hRpn2 (940–953) or hRpn2 (944-953) was injected into a calorimeter cell containing 20 or 18 μM hRpn13 Pru, respectively. The binding isotherms (top) were integrated to yield the change in enthalpy as a function of hRpn2 peptide addition. The data fit well to a 1-site binding mode with the indicated thermodynamic values. (**b**) Graphs of secondary structure (upper panel) and order parameter S^2 (lower panel) for hRpn2-bound hRpn13 (1-150) calculated by TALOS+ (http//spin.niddk.nih.gov/bax/software/TALOS/) based on HN, Cα, Cβ, CO and N chemical shift assignments.

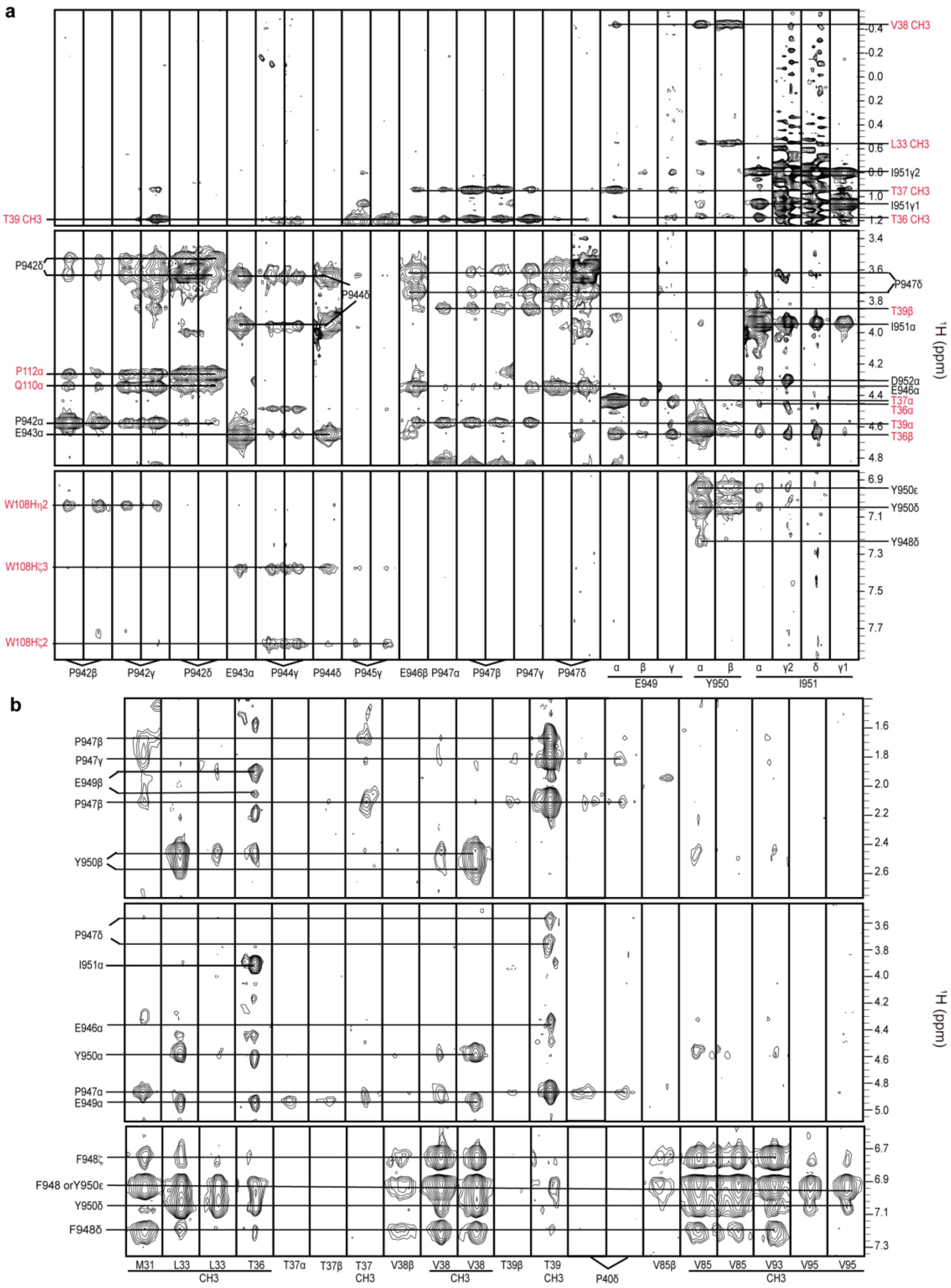

Supplementary Figure 2: NOE data define the hRpn13 Pru-hRpn2 contact surface. (**a**) Selected regions from a ^{1}H, ^{13}C edited NOESY experiment acquired with ^{15}N, ^{13}C-labeled hRpn2 (940-953) and equimolar unlabeled hRpn13 Pru. Intermolecular and intramolecular NOEs are indicated by red and black labels, respectively. (**b**) Selected regions from a ^{1}H, ^{13}C half-filtered NOESY experiment acquired with ^{15}N, ^{13}C-labeled hRpn13 Pru and equimolar unlabeled hRpn2 (940-953).

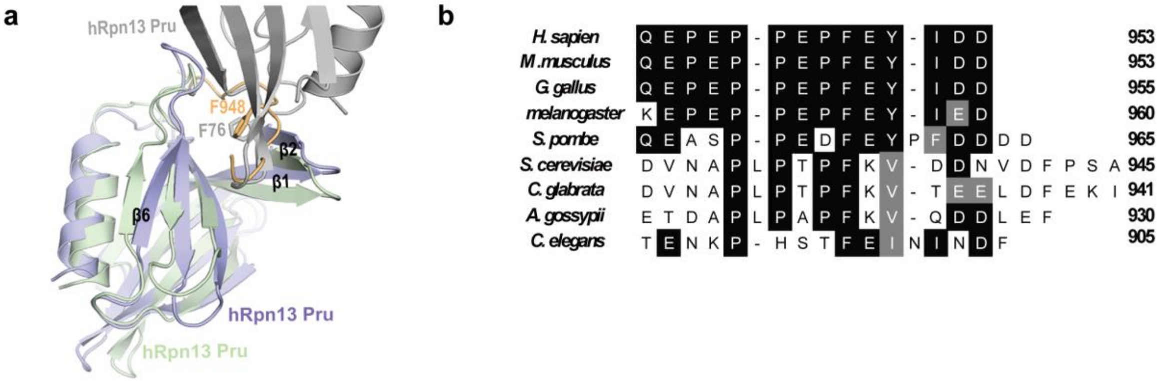

Supplementary Figure 3: Comparison of hRpn13 Pru from a crystal structure and with hRpn2 bound. (**a**) Overlay of hRpn13 Pru in its free crystal form (light green, PDB 5IRS) and the NMR structure of hRpn2-bound hRpn13 Pru (periwinkle blue). In the crystal another hRpn13 Pru molecule (shown in grey) packs F76 into the location of hRpn2 F948 (orange). (**b**) Sequence alignment across species for the C-terminal amino acids of Rpn2, generated by using Clustal Omega (http://www.ebi.ac.uk/Tools/msa/clustalo/). Strictly and moderately conserved amino acids are shaded in black and grey respectively.

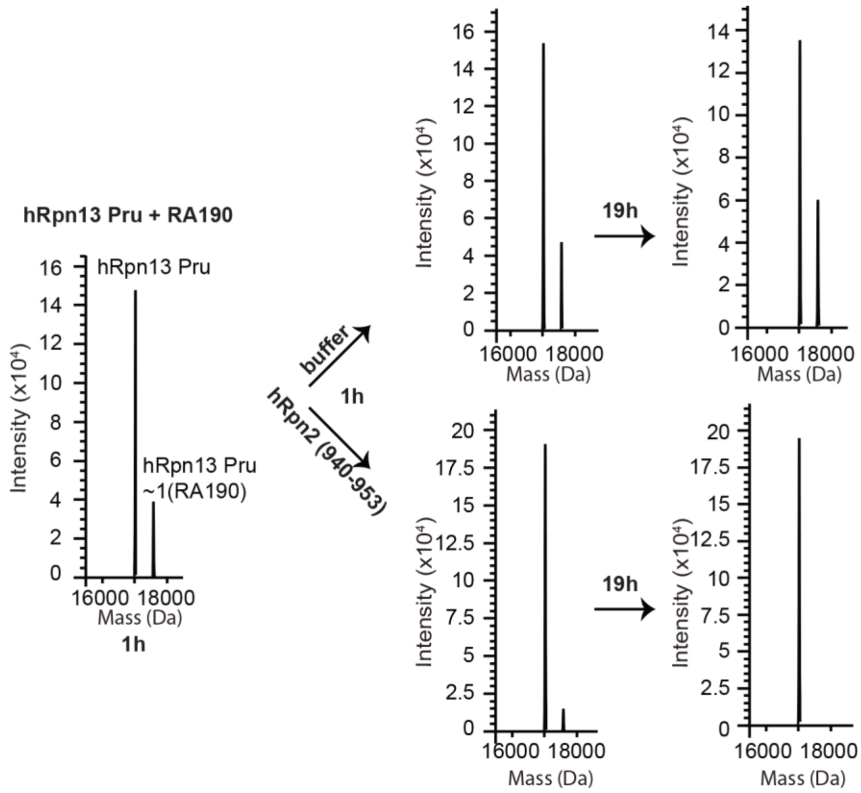

Supplementary Figure 4: RA190 conjugation to hRpn13 Pru is labile. 2 μM hRpn13 Pru was incubated with 20 μM RA190 for one hour and then subjected to LC-MS analysis. A parallel sample was then split and incubated for 1 or 19 hours with 10-fold molar excess hRpn2 (940-953) or equivalent volume of buffer for a final concentration of 2 μM hRpn13 Pru and 20 μM RA190 with or without 20 μM hRpn2. The four samples were subjected to LC-MS analysis.

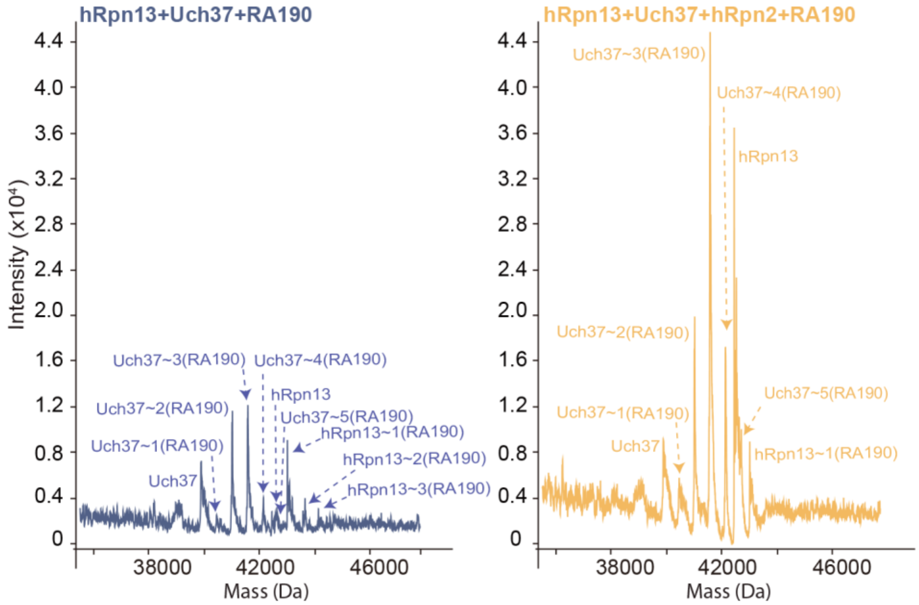

Supplementary Figure 5: RA190 adducts to Uch37. hRpn13 and Uch37 each at 2 µM (blue) or also with equimolar hRpn2 (940-953) (orange) was incubated with 50 µM RA190 for 2 hours and the samples loaded onto an Agilent 6520 Accurate-Mass Q-TOF LC/MS System. Analyses were performed by Mass Hunter Workstation (version B.06.01).

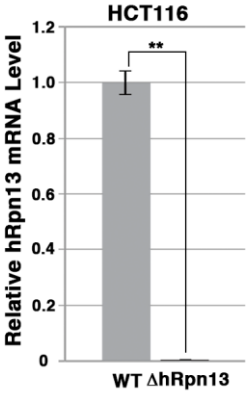

Supplementary Figure 6: hRpn13 loss was confirmed by qPCR in hRpn13-deleted (ΔhRpn13) HCT 116 cells. Total RNA from WT or ΔhRpn13 cells was purified and subjected to quantitative real-time PCR for hRpn13 mRNA analysis. GAPDH was used as an internal standard. Excel was used to quantify six independent experiments with the standard error of the mean (s.e.m.) shown. **, $p<0.05$ as determined by a Student's *t*-test (two tails, two-sample equal variance).

1c

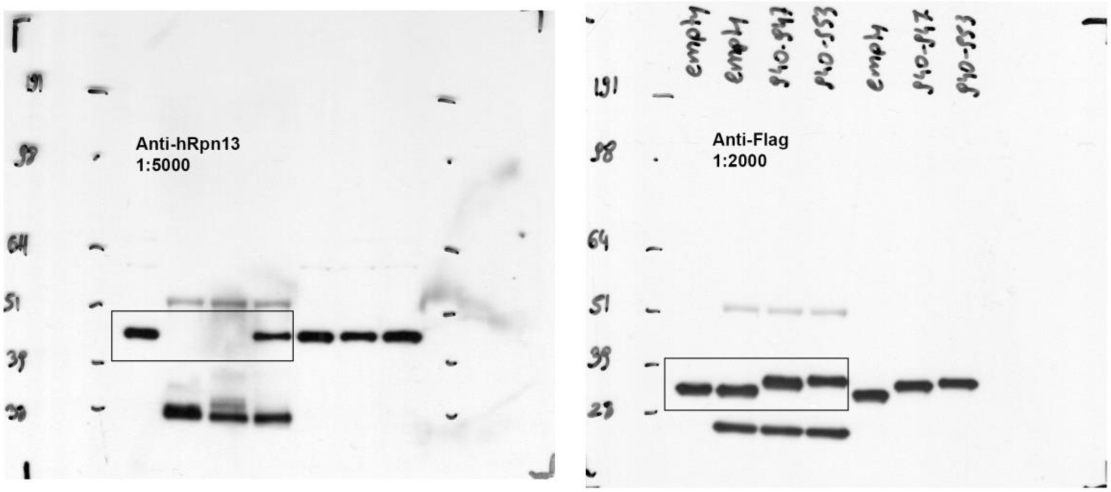

3g

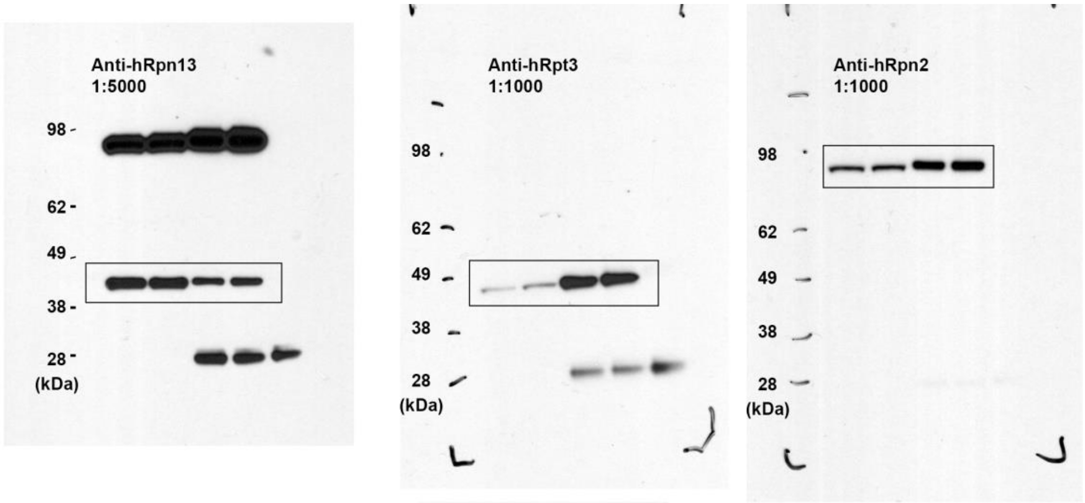
Anti-hRpn13
1:5000
98
62
49
38
28
(kDa)
Anti-hRpt3
1:1000
98
62
49
38
28
(kDa)
Anti-hRpn2
1:1000
98
62
49
38
28
(kDa)

3h

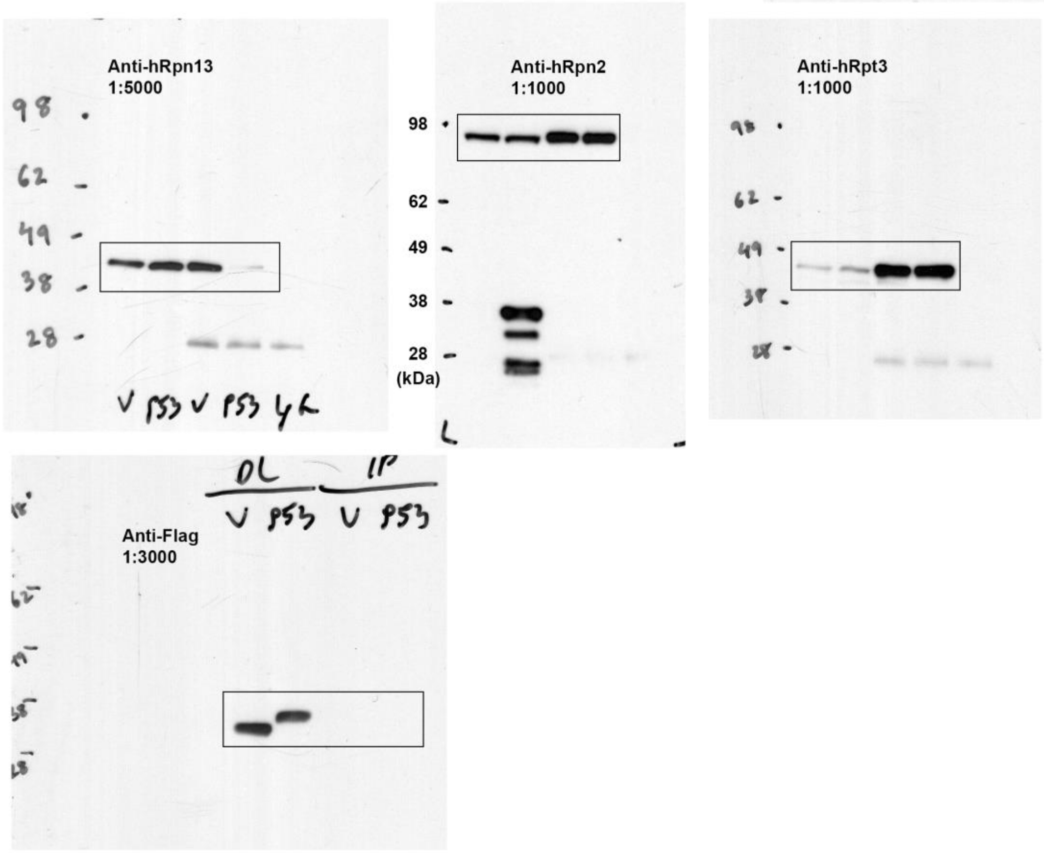
Anti-hRpn13
1:5000
98
62
49
38
28
Anti-hRpn2
1:1000
98
62
49
38
28
(kDa)
Anti-hRpt3
1:1000
98
62
49
38
28
Anti-Flag
1:3000

4a

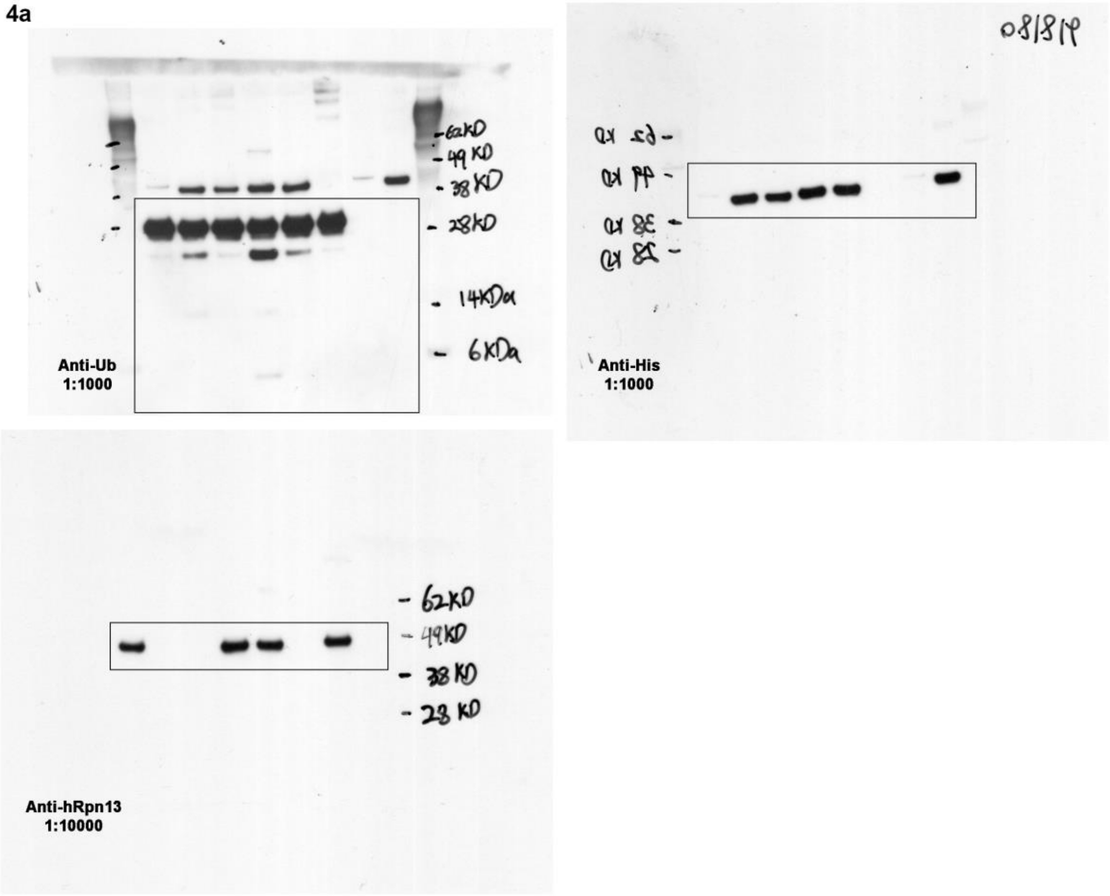
62kD
49 KD
38 KD
28kD
14kDa
6kDa
Anti-Ub
1:1000
Anti-His
1:1000
62KD
49KD
38 KD
28 KD
Anti-hRpn13
1:10000

5a

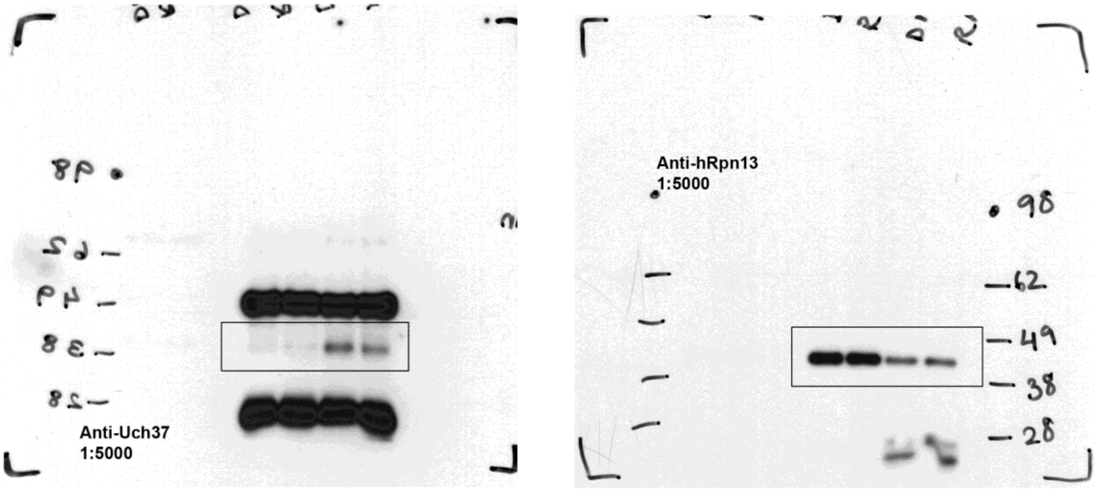

5b

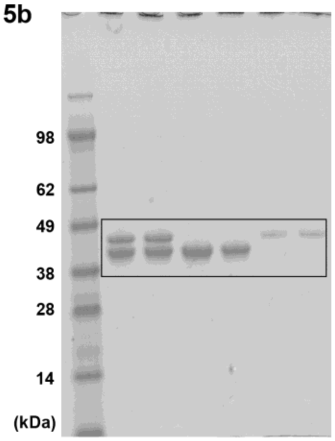

5c:hRpn13

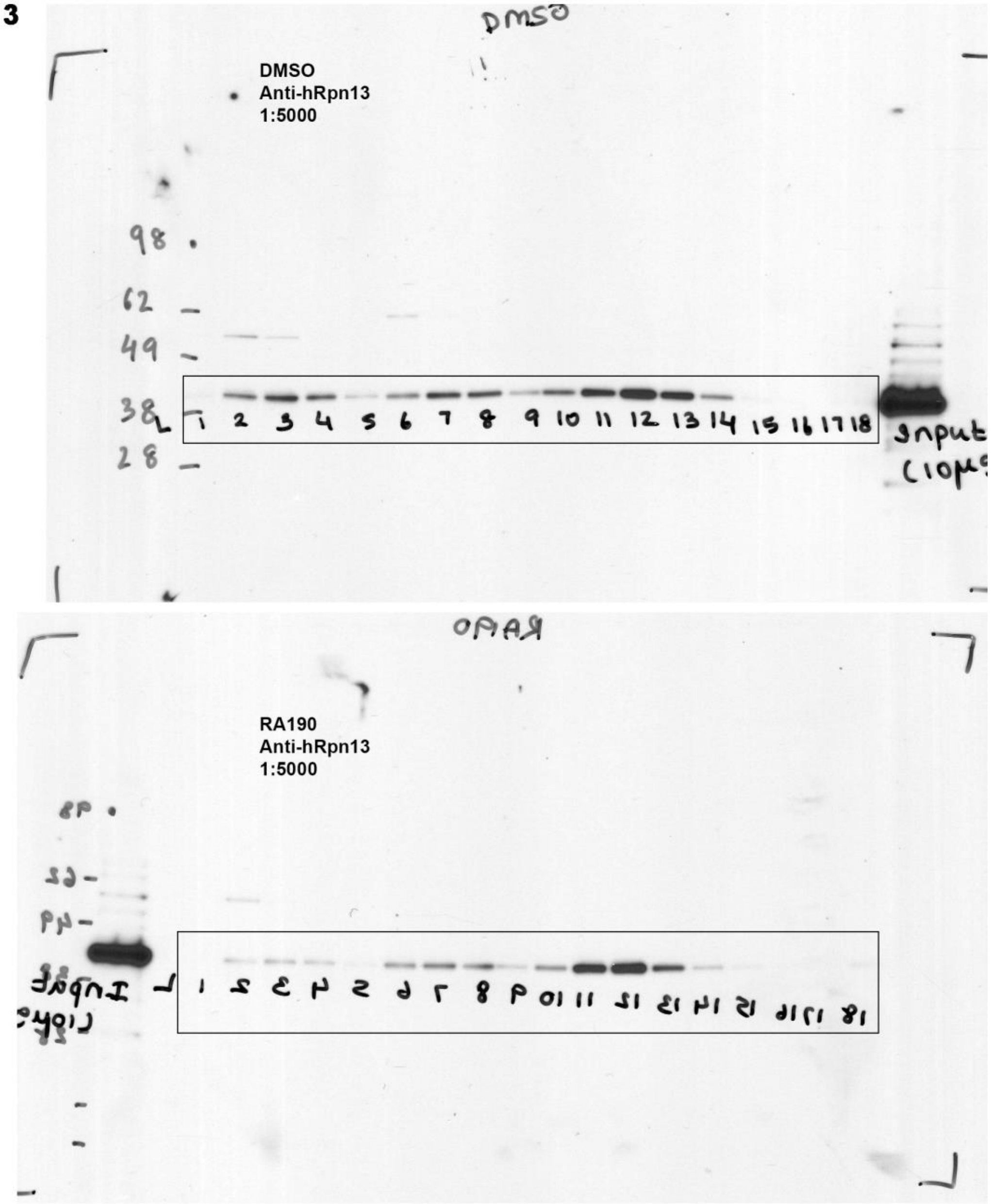
DMSO
DMSO
Anti-hRpn13
1:5000
98
62
49
38
28
1 2 3 4 5 6 7 8 9 10 11 12 13 14 15 16 17 18
Input
RA190
Anti-hRpn13
1:5000

5c:Uch37

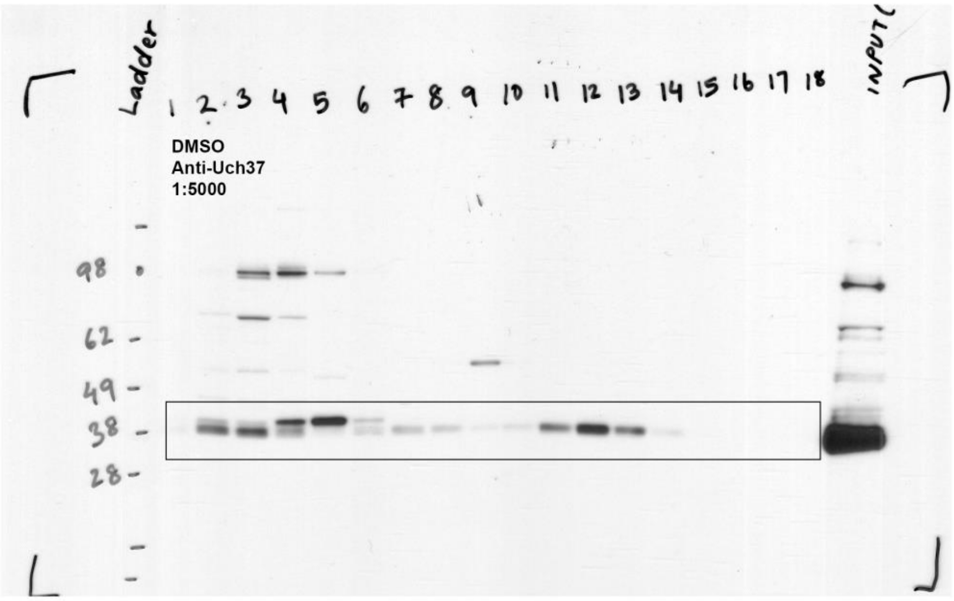

Ladder
1 2 3 4 5 6 7 8 9 10 11 12 13 14 15 16 17 18
INPUT
DMSO
Anti-Uch37
1:5000
98
62
49
38
28

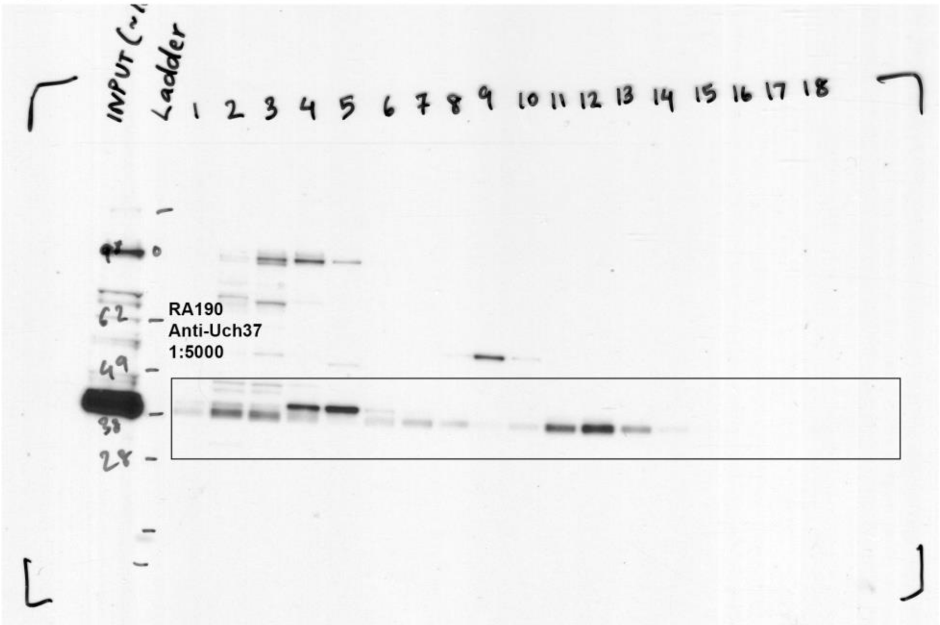

INPUT
Ladder
1 2 3 4 5 6 7 8 9 10 11 12 13 14 15 16 17 18
RA190
Anti-Uch37
1:5000
62
49
38
28

5c:β5

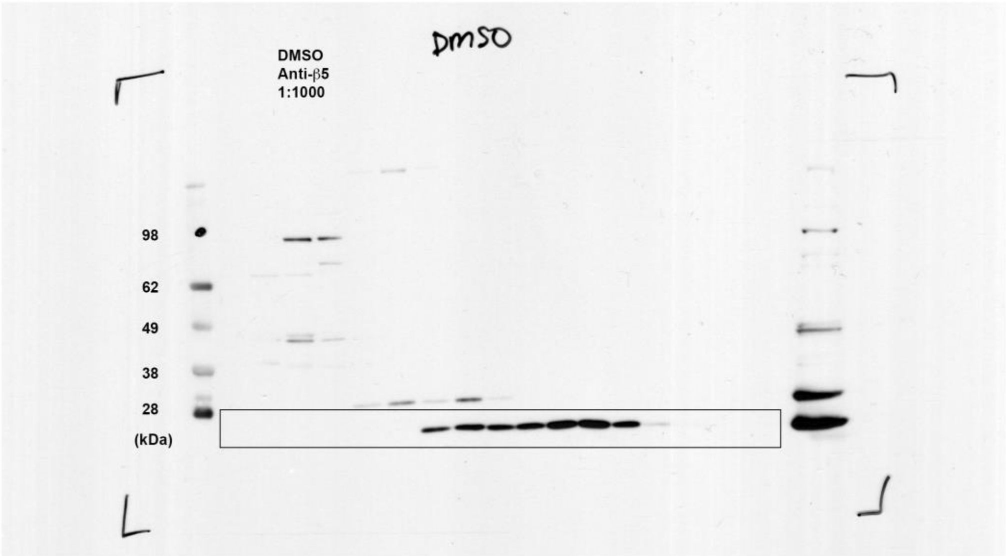
DMSO
DMSO
Anti-β5
1:1000
98
62
49
38
28
(kDa)

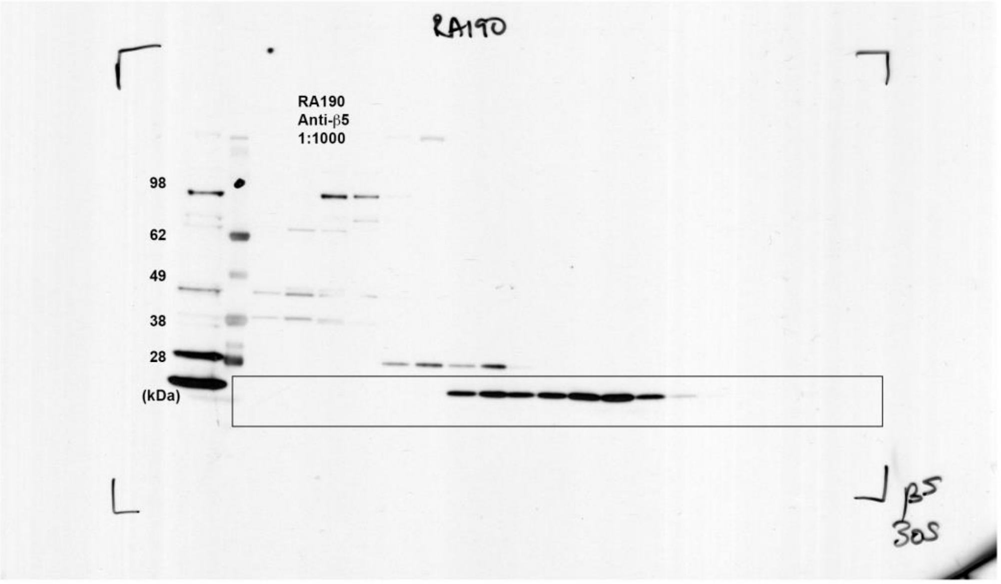
RA190
RA190
Anti-β5
1:1000
98
62
49
38
28
(kDa)
β5
30S

5c:INO80A

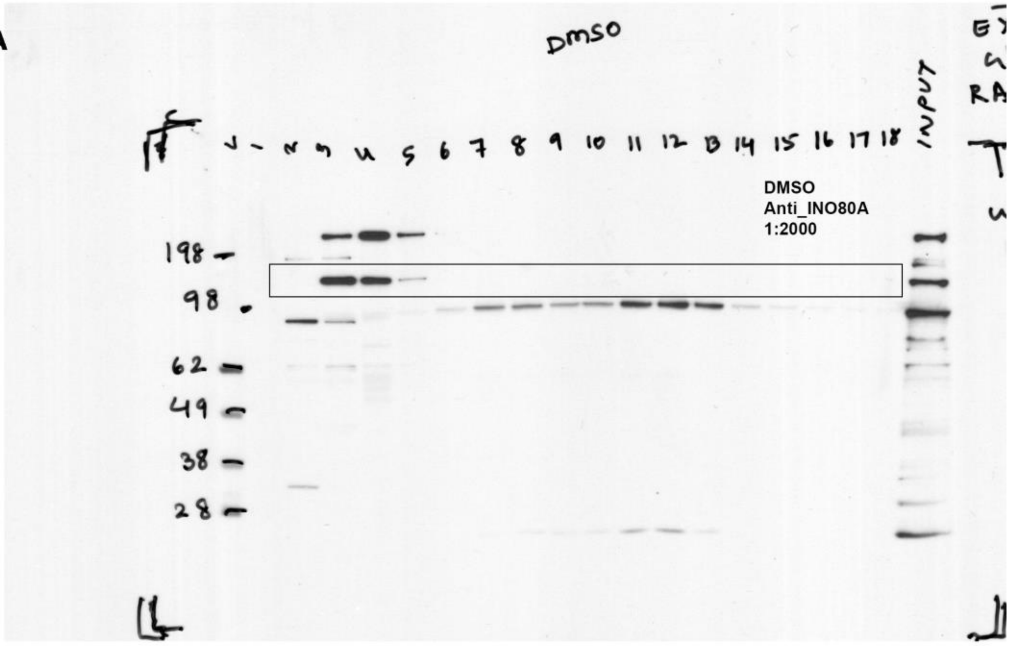

DMSO
Anti_INO80A
1:2000
198
98
62
49
38
28
INPUT

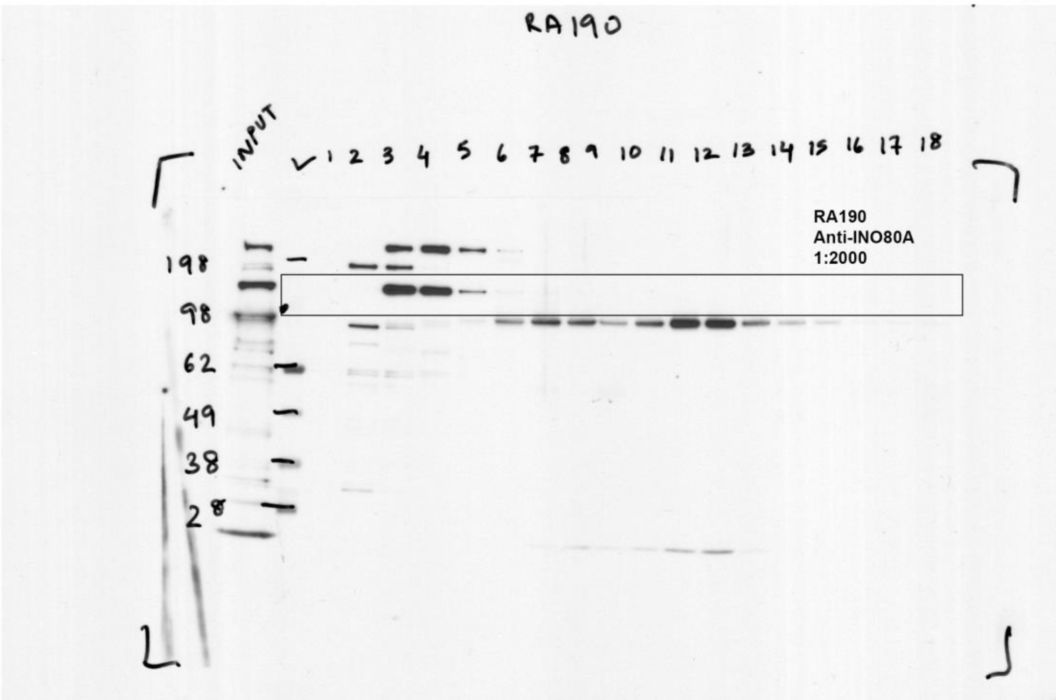

RA190
Anti-INO80A
1:2000
INPUT
198
98
62
49
38
28

5c:β actin

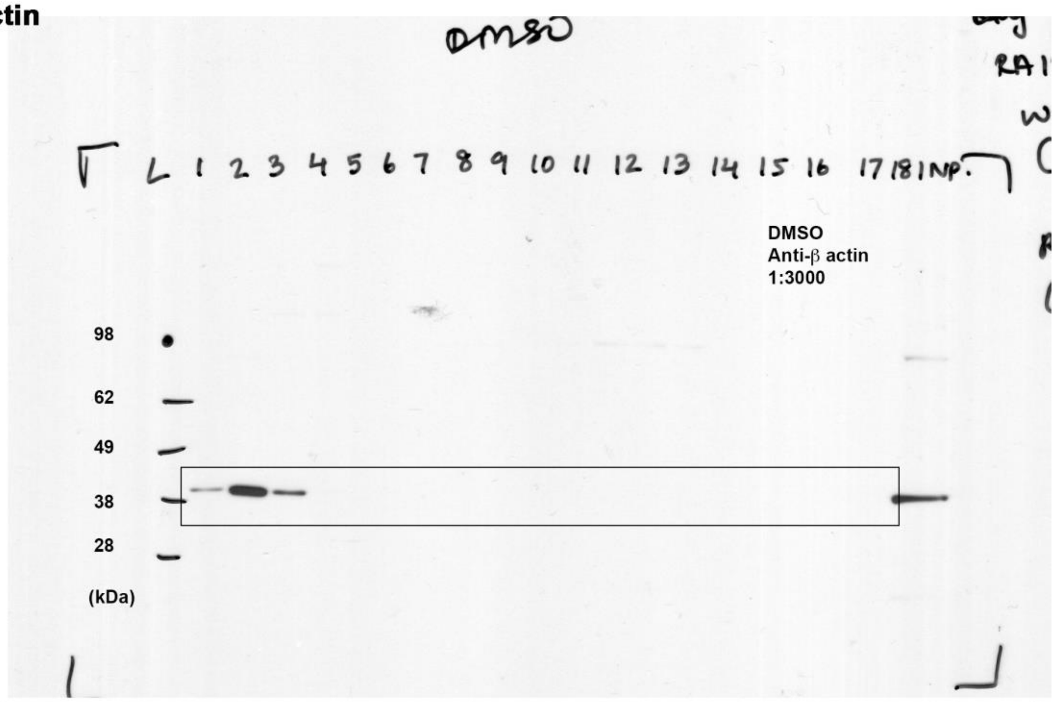

DMSO
L 1 2 3 4 5 6 7 8 9 10 11 12 13 14 15 16 17 18 INP.
DMSO
Anti-β actin
1:3000
98
62
49
38
28
(kDa)

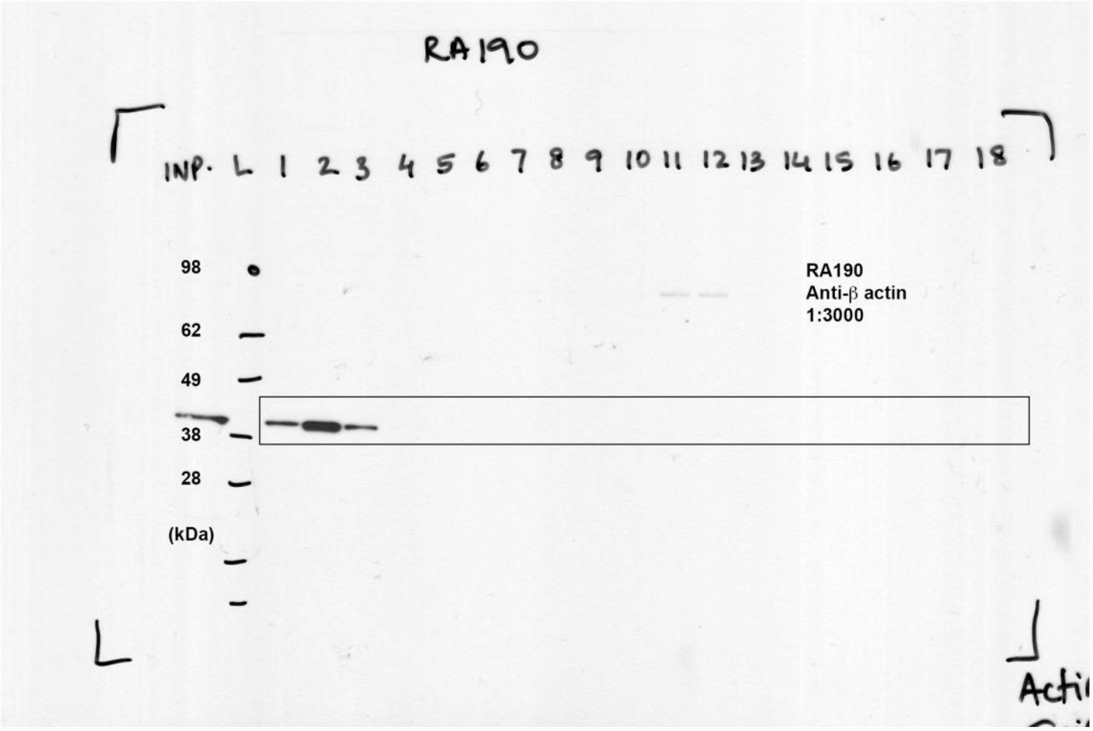

RA190
INP. L 1 2 3 4 5 6 7 8 9 10 11 12 13 14 15 16 17 18
RA190
Anti-β actin
1:3000
98
62
49
38
28
(kDa)

5d

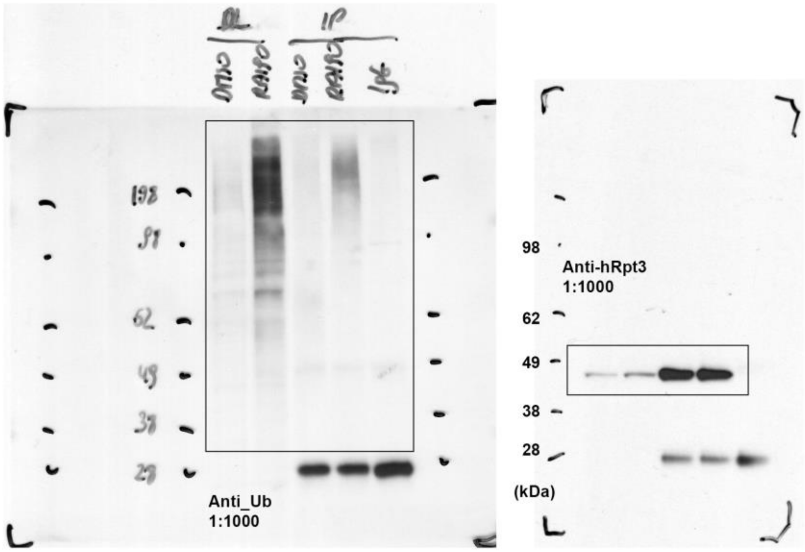

192
97
62
49
38
28
Anti_Ub
1:1000
98
Anti-hRpt3
1:1000
62
49
38
28
(kDa)

5e

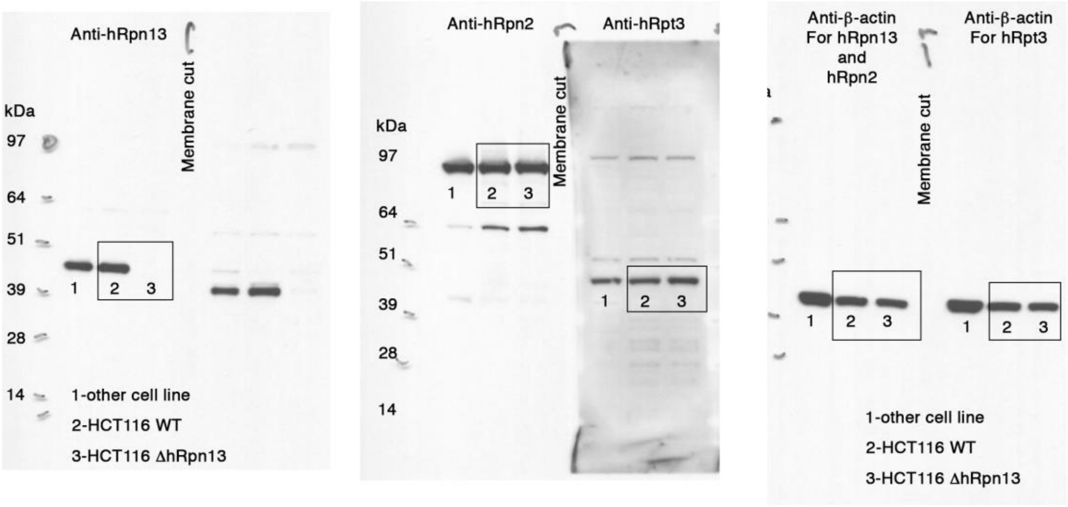

Anti-hRpn13
Membrane cut
kDa
97
64
51
39
28
14
1 2 3
1-other cell line
2-HCT116 WT
3-HCT116 ΔhRpn13
Anti-hRpn2
Anti-hRpt3
Membrane cut
kDa
97
64
51
39
28
14
1 2 3
1 2 3
Anti-β-actin
For hRpn13
and
hRpn2
Anti-β-actin
For hRpt3
Membrane cut
1 2 3
1 2 3
1-other cell line
2-HCT116 WT
3-HCT116 ΔhRpn13

5f

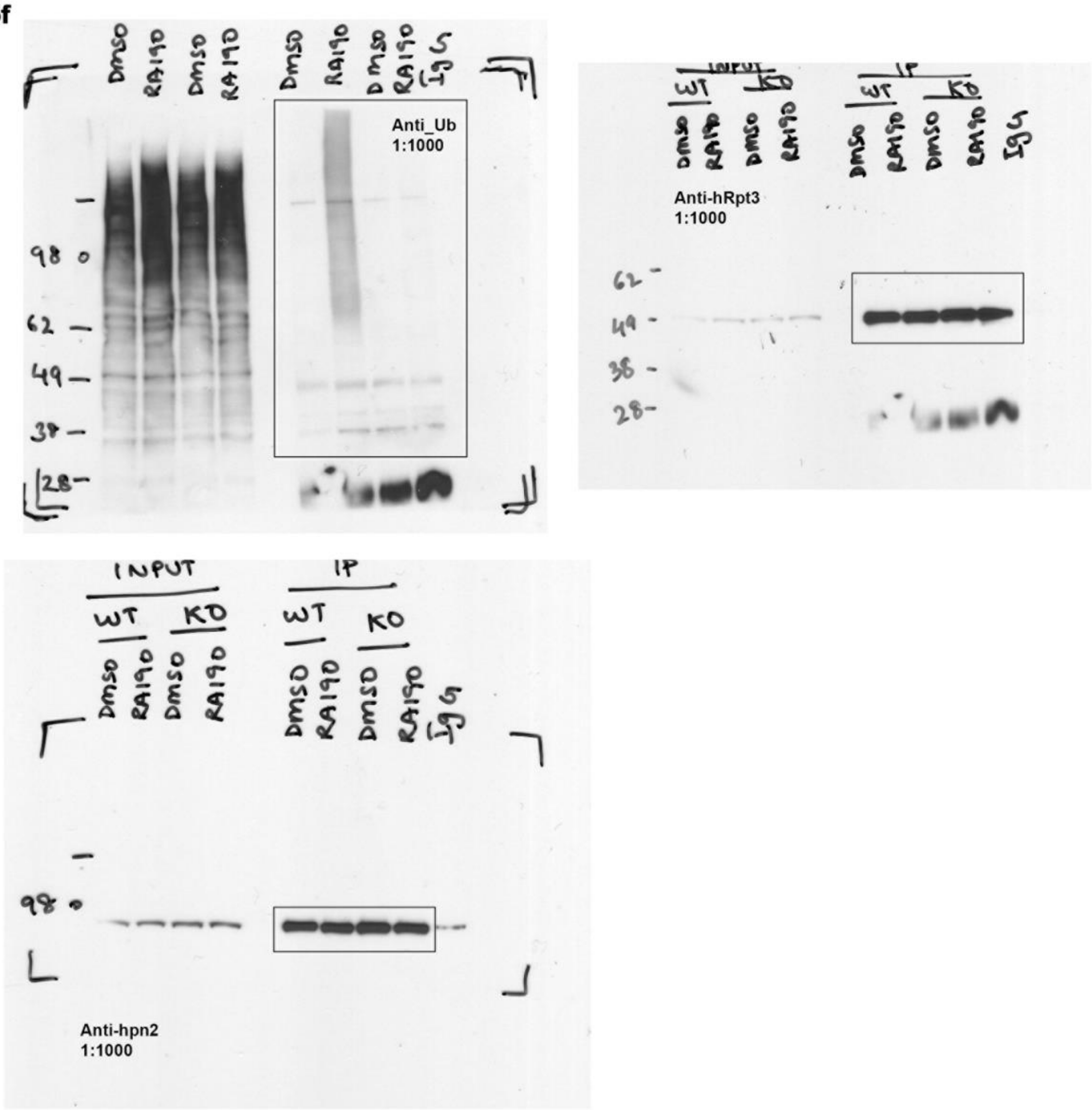

Supplementary Figure 7: uncropped images for figure 1c, 3g,h, 4a and 5a,b,c,d,e,f.

Supplementary Table 1: Observed NOE interactions between the hRpn13 Pru domain and hRpn2.

**hRpn13 Pru**	**hRpn2 (940-953)**
M31 (methyl)	P947 (Hα) and F948 (Hδ#, Hε#, Hζ)
L33 (methyl)	F948 (HN), E949 (HN, Hα) and Y950 (HN, Hα, Hβ#, Hδ#, Hε#)
T36 (Hα)	Y950 (Hδ#), I951 (Hα)
T36 (Hβ)	E949 (Hγ#), I951 (Hα, Hδ1#, Hγ2#),
T36 (methyl)	E949 (Hα, Hγ#), I951 (HN, Hα, Hδ1#, Hγ1#, Hγ2#), Y950 (HN, Hα, Hβ#, Hδ#, Hε#), D952 (HN)
T37 (Hα)	F948 (HN), E949 (HN, Hα, Hβ#, Hγ#), Y950 (HN)
T37 (Hβ)	F948 (HN), E949 (Hα)
T37 (methyl)	P947 (Hα, Hβ#, Hγ#), F948 (HN), E949 (HN, Hα, Hγ#)
V38 (HN)	F948 (HN), E949 (HN, Hα), Y950 (HN)
V38 (Hβ)	F948 (HN, Hδ#, Hε#, Hζ), E949 (HN), Y950 (HN)
V38 (methyl)	F948 (HN, Hδ#, Hε#, Hζ), E949 (HN, Hα), Y950 (HN, Hα, Hβ#, Hδ#, Hε#)
T39 (Hα)	E946 (HN), P947 (Hα, Hβ#, Hγ#)
T39 (Hβ)	E946 (HN), P947 (Hα, Hβ#, Hγ#)
T39 (methyl)	E946 (Hα), P947 (Hα, Hβ#, Hγ#, Hδ#), F948 (HN)
P40 (Hβ#)	P945 (Hγ#)
P40 (Hδ#)	P945 (Hγ#, Hδ#), P947 (Hα, Hβ#, Hγ#)
V85 (Hβ)	F948 (Hε#, Hζ)
V85 (methyl)	F948 (Hδ#, Hε#, Hζ), Y950 (Hδ#, Hε#)
V93 (methyl)	F948 (Hδ#, Hε#, Hζ)
V95 (methyl)	Y950 (Hδ#, Hε#)
R104 (Hγ#)	Y950 (Hε#)
R104 (Hδ#)	Y950 (Hε#)
W108 (Hζ2)	P944 (Hγ#), P945 (Hγ#)
W108 (Hζ3)	E943 (HN, Hα), P944 (Hγ#, Hδ#), P945 (Hδ#)
W108 (Hε3)	P945 (Hδ#)
W108 (Hη2)	P944 (Hβ#, Hγ#, Hδ#), E943 (HN)
E110 (Hα)	P942 (Hα, Hβ#, Hγ#)
P112 (Hα)	P942 (Hα, Hβ#, Hγ#, Hδ#)
P112 (Hγ#)	P942 (Hβ#)

#: pseudo atoms lacking stereo assignment

Supplementary Table 2: LC-MS data for mixtures of hRpn13, hRpn2, and RA190.

Sample	hRpn13 species			
	Free	1(RA190)	2(RA190)	3(RA190)
Pru	17017.49	17577.89	18138.15	ND
Pru+hRpn2	17017.50	ND	ND	ND
DEUBAD	16323.12	16881.97	ND	ND
hRpn13	42450.64	43012.33	43572.64	44134.00
hRpn13+hRpn2	42451.88	43012.66	ND	ND

ND = not detected

Table listing the molecular weight and indicated species from LC-MS analyses of listed samples. 2 μM free protein or equimolar mixture was incubated with 10-fold molar excess RA190 for 2 hours at 4 °C and the samples subjected to LC-MS. The molecular weight of RA190 is 560.30 Da and the species indicated are based on calculated molecular weight. Molecular weight was calculated by OpenLAB CDS ChemStation Edition C.01.05 or Mass Hunter Workstation (version B.06.01). ND, not detected. 1(RA190), hRpn13 with one RA190 molecule adducted; 2(RA190), hRpn13 with two RA190 molecules adducted; 3(RA190), hRpn13 with three RA190 molecules adducted.

Supplementary Table 3: LC-MS data for mixtures of Uch37, hRpn13, hRpn2, and RA190.

Species	Sample		
	Uch37	hRpn13+Uch37	hRpn13+Uch37+hRpn2
Uch37	39906.16	39903.53	39905.29
Uch37~1(RA190)	40452.51	40453.00	40453.00
Uch37~2(RA190)	41022.61	41025.84	41024.85
Uch37~3(RA190)	41583.98	41586.44	41585.38
Uch37~4(RA190)	42146.80	42143.98	42133.95
Uch37~5(RA190)	42707.82	42705.00	42707.70
hRpn13	NA	42450.49	42452.39
hRpn13~1(RA190)	NA	43013.32	43013.43
hRpn13~2(RA190)	NA	43572.10	ND
hRpn13~3(RA190)	NA	44132.39	ND

NA = not applicable; ND = not detected

Table listing the molecular weight and indicated species from LC-MS analyses of listed samples. 2 μM Uch37, equimolar mixture of 2 μM hRpn13 and Uch37, or equimolar mixture of 2 μM hRpn13, Uch37, and hRpn2 was incubated with 25-fold molar excess RA190 for 2 hours at 4 °C and then subjected to LC-MS. Molecular weight for indicated species were calculated by Mass Hunter Workstation (version B.06.01) (NA, not applicable; ND, not detected). 1(RA190), hRpn13 or Uch37 with one RA190 molecule adducted; 2(RA190), hRpn13 or Uch37 with two RA190 molecules adducted; 3(RA190), hRpn13 or Uch37 with three RA190 molecules adducted; 4(RA190), Uch37 with four RA190 molecules adducted; 5(RA190), Uch37 with five RA190 molecules adducted.

Supplementary Table 4: Oligonucleotides for sgRNA targeting the *ADRM1* gene.

		Primer Sequences (5' to 3')	Obtained clones
sgRNA-B	sense	AAACTGCGATTCGGTTAGGAACTTC	B11
	antisense	CACCGAAGTTCCTAACCGAATCGCA	
sgRNA-C	sense	AAACATTGCGGTGTGAAATTTCCAC	C8, C9
	antisense	CACCGTGGAAATTTCACACCGCAAT	
