# Supplementary figures and images for: Structure of the Rpn13-Rpn2 complex provides insights for Rpn13 and Uch37 as anticancer targets
Source: Nat Commun. 2017 Jun 9;8:15540. doi: 10.1038/ncomms15540 (PMC5494190; doi:10.1038/ncomms15540)

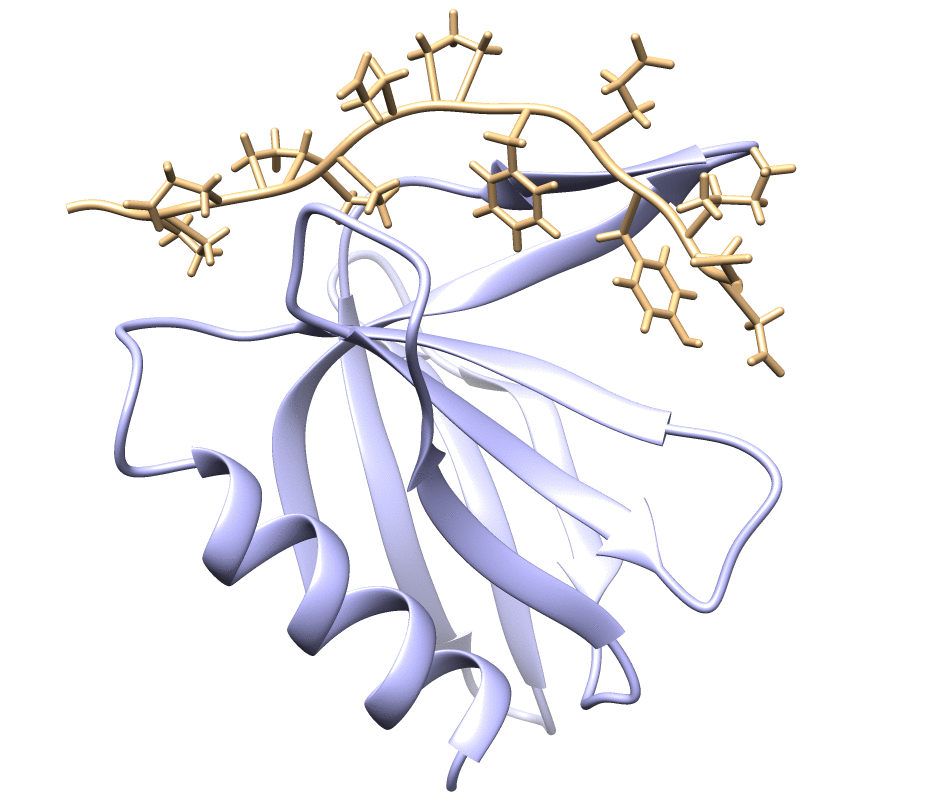

Supplement: Supplementary Movie 1 — Comparison of the hRpn13 Pru domain when bound to hRpn2 (940-953) with its free crystal structure. The backbone atoms of hRpn13 Pru domain from the free crystal structure (PDB 5IRS) was superimposed onto that of hRpn13 Pru bound to hRpn2 (940-953) in Pymol (PyMOL Molecular Graphics System, http://www.pymol.org) and the coordinates written to PDB files for import into UCSF Chimera. 61 frames were generated by using the "morph conformations" feature in UCSF Chimera to generate a visual representation of the conformational rearrangements within the hRpn13 Pru domain that begins with its free crystallized state and ends with its hRpn2-bound configuration. β1, β2, and β6 of hRpn13 Pru reconfigure to bend towards hRpn2 for optimized interactions. hRpn13 Pru is displayed in periwinkle blue and hRpn2 (940-953) in orange. [file ncomms15540-s2.tif]
